# Tetra­aqua­bis(biuret-κ^2^ *O*,*O*′)gadolinium(III) trichloride

**DOI:** 10.1107/S1600536808008660

**Published:** 2008-04-02

**Authors:** William T. A. Harrison

**Affiliations:** a Department of Chemistry, University of Aberdeen, Meston Walk, Aberdeen AB24 3UE, Scotland

## Abstract

In the title compound, [Gd(C_2_H_5_N_3_O_2_)_2_(H_2_O)_4_]Cl_3_, which is isostrucutural with its yttrium analogue, the Gd^3+^ ion (site symmetry 2) is bonded to eight O atoms (arising from two *O*,*O*′-bidentate biuret mol­ecules and four water mol­ecules) in a distorted square-anti­prismatic arrangement. A network of N—H⋯O, N—H⋯Cl and O—H⋯Cl hydrogen bonds helps to establish the packing, leading to a three-dimensional network. One of the chloride ions has site symmetry 2.

## Related literature

For related structures, see: Haddad (1987 ▶, 1988 ▶); Harrison (2008 ▶). For related literature, see: Bernstein *et al.* (1995 ▶). For valence-sum calculations, see: Brese & O’Keeffe (1991 ▶).
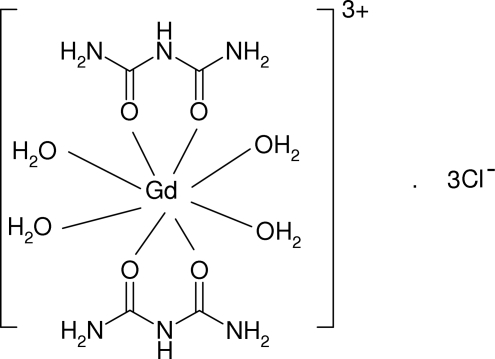


## Experimental

### 

#### Crystal data

[Gd(C_2_H_5_N_3_O_2_)_2_(H_2_O)_4_]Cl_3_
*M* *_r_* = 541.84
Monoclinic, 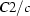

*a* = 7.6501 (3) Å
*b* = 13.2164 (5) Å
*c* = 17.4557 (6) Å
β = 100.961 (1)°
*V* = 1732.69 (11) Å^3^
*Z* = 4
Mo *K*α radiation
μ = 4.33 mm^−1^
*T* = 293 (2) K
0.47 × 0.34 × 0.06 mm

#### Data collection

Bruker SMART1000 CCD diffractometer
Absorption correction: multi-scan (*SADABS*; Bruker, 1999 ▶) *T*_min_ = 0.235, *T*_max_ = 0.781
8649 measured reflections
3134 independent reflections
2902 reflections with *I* > 2σ(*I*)
*R*_int_ = 0.025

#### Refinement

*R*[*F* ^2^ > 2σ(*F* ^2^)] = 0.033
*wR*(*F* ^2^) = 0.088
*S* = 1.06
3134 reflections
101 parameters
H-atom parameters constrained
Δρ_max_ = 2.99 e Å^−3^
Δρ_min_ = −3.45 e Å^−3^

### 

Data collection: *SMART* (Bruker, 1999 ▶); cell refinement: *SAINT* (Bruker, 1999 ▶); data reduction: *SAINT*; program(s) used to solve structure: *SHELXS97* (Sheldrick, 2008 ▶); program(s) used to refine structure: *SHELXL97* (Sheldrick, 2008 ▶); molecular graphics: *ORTEP-3* (Farrugia, 1997 ▶); software used to prepare material for publication: *SHELXL97*.

## Supplementary Material

Crystal structure: contains datablocks I, global. DOI: 10.1107/S1600536808008660/sg2230sup1.cif

Structure factors: contains datablocks I. DOI: 10.1107/S1600536808008660/sg2230Isup2.hkl

Additional supplementary materials:  crystallographic information; 3D view; checkCIF report

## Figures and Tables

**Table 1:** Selected bond lengths (Å)

Gd1—O1	2.350 (2)
Gd1—O2	2.375 (2)
Gd1—O4	2.407 (3)
Gd1—O3	2.414 (2)

**Table 2:** Hydrogen-bond geometry (Å, °)

*D*—H⋯*A*	*D*—H	H⋯*A*	*D*⋯*A*	*D*—H⋯*A*
N1—H1⋯O1^i^	0.86	2.10	2.910 (4)	157
N1—H2⋯Cl1^ii^	0.86	2.40	3.194 (3)	154
N2—H3⋯Cl1^ii^	0.86	2.53	3.315 (3)	153
N3—H4⋯Cl1^iii^	0.86	2.54	3.363 (3)	161
N3—H5⋯Cl1^iv^	0.86	2.53	3.311 (3)	151
O3—H6⋯Cl1^v^	0.81	2.39	3.163 (2)	162
O3—H7⋯Cl2^vi^	0.79	2.28	3.059 (2)	167
O4—H8⋯Cl1	0.76	2.45	3.208 (2)	178
O4—H9⋯Cl2	0.80	2.41	3.127 (2)	150

Symmetry codes: (i) 
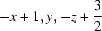
; (ii) 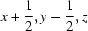; (iii) 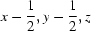; (iv) 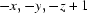; (v) 
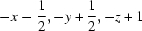
; (vi) 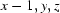.

**Fig. 1.**
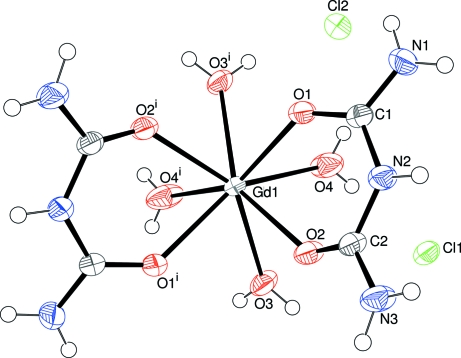
View of the molecular structure of (I) showing 50% displacement ellipsoids (arbitrary spheres for the H atoms). Symmetry code: (i) -x, y, 3/2 - z.

## supplementary crystallographic information

### Comment

The title compound, (I), is isostructural with its recently described yttrium-containing analogue (Harrison, 2008).

The complete [Gd(biur)_2_(H_2_O)_4_]^3+^ (biur = biuret, C_2_H_5_N_3_O_2_) complex ion in (I) is generated by crystallographic 2-fold symmetry, with the metal atom lying on the rotation axis. Two uncoordinated chloride ions, one of which has site symmetry 2, complete the structure (Fig. 1) of (I).

The resulting GdO_8_ polyhedral geometry in (I) (Table 1) is a distorted square antiprism. The nominal square face formed by atoms O1, O2, O1^i^ and O2^i^ (i = -*x*, *y*, 3/2 - *z*) is reasonably regular, but the second face formed by the four water molecules (O3, O4, O3^i^ and O4^i^) is much more distorted, and the diagonal O3···O3^i^ O4···O4^i^ distances of 4.322 (4)Å and 3.551 (4) Å, respectively, are very different. Gd1 deviates from the mean planes of O1/O2/O1^i^/O2^i^ and O3/O4/O3^i^/O4^i^ by 1.1542 (16)Å and 1.3501 (19) Å, respectively. The gadolinium bond valence sum of 2.90, calculated by the Brese & O'Keffe (1991) method, indicates that it is slightly underbonded in (I), whereas in [Y(biur)_2_(H_2_O)_4_].3Cl (Harrison, 2008), the yttrium cation was distinctly overbonded with a BVS of 3.34 (expected value = 3.00 in both cases).

The dihedral angle betwen the N1/C1/O1/N2 and N2/C2/O2/N3 fragments of the biuret molecule is 21.2 (2)°, which is far larger than the equivalent value of 5.06 (10)° in [Y(biur)_2_(H_2_O)_4_].3Cl (Harrison, 2008). The gadolinium cation in (I) deviates from the N1/C1/O1/N2 and N2/C2/O2/N3 mean planes by -0.834 (6)Å and 0.530 (6) Å, respectively, indicating that the six-membered chelate ring is non-planar.

The component species in (I) are linked by a dense array of N—H···O, N—H···Cl and O—H···Cl hydrogen bonds (Table 2) resulting in a three-dimensional network. Of note is the N—H···O hydrogen bond, which results in [100] chains of cations, linked by *R*^2^_2_(8) loops (Bernstein *et al.*, 1995), as also seen in the yttrium phase (Harrison, 2008).

For related rare-earth–biuret complexes, see: Haddad (1987 and 1988).

### Experimental

0.1 *M* Aqueous solutions of GdCl_3_ (10 ml) and biuret (10 ml) were mixed and a small quantity of dilute hydrochloric acid was added, to result in a colourless solution. Colourless blocks of (I) grew over several days as the water slowly evaporated.

### Refinement

The crystal quality was rather poor, with smeared and split peaks evident in the diffraction pattern.

The N-bound hydrogen atoms were geometrically placed (N—H = 0.88 Å) and refined as riding with *U*_iso_(H) = 1.2*U*_eq_(N). The water H atoms were located in difference maps and refined as riding in their as-found relative positions with *U*_iso_(H) = 1.2*U*_eq_(O).

The highest difference peak and deepest difference hole are 0.71Å and 0.75Å from Gd1, respectively.

### Crystal data

**Table:**

[Gd(C_2_H_5_N_3_O_2_)_2_(H_2_O)_4_]Cl_3_	*F*_000_ = 1052
*M**_r_* = 541.84	*D*_x_ = 2.077 Mg m^−^^3^
Monoclinic, *C*2/*c*	Mo *K*α radiation λ = 0.71073 Å
Hall symbol: -C 2yc	Cell parameters from 6423 reflections
*a* = 7.6501 (3) Å	θ = 2.4–32.5º
*b* = 13.2164 (5) Å	µ = 4.33 mm^−^^1^
*c* = 17.4557 (6) Å	*T* = 293 (2) K
β = 100.961 (1)º	Slab, colourless
*V* = 1732.69 (11) Å^3^	0.47 × 0.34 × 0.06 mm
*Z* = 4	

### Data collection

**Table:**

Bruker SMART1000 CCD diffractometer	3134 independent reflections
Radiation source: fine-focus sealed tube	2902 reflections with *I* > 2σ(*I*)
Monochromator: graphite	*R*_int_ = 0.025
*T* = 293(2) K	θ_max_ = 32.5º
ω scans	θ_min_ = 2.4º
Absorption correction: multi-scan(SADABS; Bruker, 1999)	*h* = −11→9
*T*_min_ = 0.235, *T*_max_ = 0.781	*k* = −19→18
8649 measured reflections	*l* = −19→26

### Refinement

**Table:**

Refinement on *F*^2^	Secondary atom site location: difference Fourier map
Least-squares matrix: full	Hydrogen site location: difmap (O-H) and geom (N-H)
*R*[*F*^2^ > 2σ(*F*^2^)] = 0.033	H-atom parameters constrained
*wR*(*F*^2^) = 0.088	*w* = 1/[σ^2^(*F*_o_^2^) + (0.0654*P*)^2^] where *P* = (*F*_o_^2^ + 2*F*_c_^2^)/3
*S* = 1.06	(Δ/σ)_max_ < 0.001
3134 reflections	Δρ_max_ = 2.99 e Å^−^^3^
101 parameters	Δρ_min_ = −3.45 e Å^−^^3^
Primary atom site location: structure-invariant direct methods	Extinction correction: none

### Special details

**Table:**

Geometry. All e.s.d.'s (except the e.s.d. in the dihedral angle between two l.s. planes) are estimated using the full covariance matrix. The cell e.s.d.'s are taken into account individually in the estimation of e.s.d.'s in distances, angles and torsion angles; correlations between e.s.d.'s in cell parameters are only used when they are defined by crystal symmetry. An approximate (isotropic) treatment of cell e.s.d.'s is used for estimating e.s.d.'s involving l.s. planes.
Refinement. Refinement of *F*^2^ against ALL reflections. The weighted *R*-factor *wR* and goodness of fit *S* are based on *F*^2^, conventional *R*-factors *R* are based on *F*, with *F* set to zero for negative *F*^2^. The threshold expression of *F*^2^ > σ(*F*^2^) is used only for calculating *R*-factors(gt) *etc*. and is not relevant to the choice of reflections for refinement. *R*-factors based on *F*^2^ are statistically about twice as large as those based on *F*, and *R*- factors based on ALL data will be even larger.

### Fractional atomic coordinates and isotropic or equivalent isotropic displacement parameters (Å^2^)

**Table:**

	*x*	*y*	*z*	*U*_iso_*/*U*_eq_	
Gd1	0.0000	0.106486 (13)	0.7500	0.02252 (7)	
Cl1	−0.04157 (10)	0.33393 (7)	0.50790 (4)	0.03672 (16)	
Cl2	0.5000	0.30307 (10)	0.7500	0.0389 (2)	
O1	0.2531 (3)	0.02702 (18)	0.71963 (13)	0.0310 (4)	
O2	−0.0822 (3)	0.01130 (17)	0.63327 (12)	0.0305 (4)	
N1	0.4595 (4)	−0.0112 (3)	0.64845 (18)	0.0434 (7)	
H1	0.5423	0.0169	0.6820	0.052*	
H2	0.4845	−0.0387	0.6072	0.052*	
N2	0.1720 (3)	−0.0598 (2)	0.60404 (15)	0.0295 (5)	
H3	0.2119	−0.0977	0.5712	0.035*	
N3	−0.1037 (4)	−0.1127 (2)	0.5433 (2)	0.0371 (6)	
H4	−0.2181	−0.1098	0.5337	0.044*	
H5	−0.0497	−0.1552	0.5186	0.044*	
C1	0.2950 (4)	−0.0121 (2)	0.66032 (16)	0.0269 (5)	
C2	−0.0103 (4)	−0.0513 (2)	0.59616 (16)	0.0265 (5)	
O3	−0.2462 (3)	0.18787 (18)	0.66543 (13)	0.0360 (5)	
H6	−0.2805	0.1748	0.6199	0.043*	
H7	−0.3244	0.2160	0.6810	0.043*	
O4	0.1191 (3)	0.2294 (2)	0.67206 (16)	0.0502 (7)	
H8	0.0833	0.2534	0.6327	0.060*	
H9	0.2181	0.2507	0.6743	0.060*	

### Atomic displacement parameters (Å^2^)

**Table:**

	*U*^11^	*U*^22^	*U*^33^	*U*^12^	*U*^13^	*U*^23^
Gd1	0.01907 (9)	0.02952 (10)	0.01740 (9)	0.000	−0.00047 (6)	0.000
Cl1	0.0337 (3)	0.0470 (4)	0.0268 (3)	0.0011 (3)	−0.0007 (2)	−0.0067 (3)
Cl2	0.0301 (4)	0.0496 (6)	0.0377 (5)	0.000	0.0081 (4)	0.000
O1	0.0217 (8)	0.0441 (12)	0.0257 (9)	0.0024 (8)	0.0006 (7)	−0.0071 (8)
O2	0.0251 (9)	0.0359 (10)	0.0275 (9)	0.0038 (8)	−0.0024 (7)	−0.0077 (8)
N1	0.0233 (11)	0.073 (2)	0.0345 (14)	−0.0075 (12)	0.0071 (10)	−0.0178 (14)
N2	0.0240 (10)	0.0363 (12)	0.0269 (11)	0.0004 (9)	0.0018 (8)	−0.0083 (9)
N3	0.0297 (13)	0.0416 (15)	0.0364 (15)	−0.0020 (9)	−0.0025 (11)	−0.0147 (11)
C1	0.0227 (11)	0.0322 (12)	0.0241 (11)	0.0010 (9)	0.0005 (9)	−0.0008 (9)
C2	0.0247 (11)	0.0296 (12)	0.0231 (11)	0.0000 (9)	−0.0010 (9)	−0.0016 (9)
O3	0.0337 (10)	0.0484 (13)	0.0228 (9)	0.0135 (9)	−0.0027 (7)	−0.0022 (9)
O4	0.0384 (13)	0.0637 (17)	0.0423 (14)	−0.0157 (12)	−0.0084 (10)	0.0257 (12)

### Geometric parameters (Å, °)

**Table:**

Gd1—O1^i^	2.350 (2)	N1—H2	0.8600
Gd1—O1	2.350 (2)	N2—C1	1.377 (4)
Gd1—O2	2.375 (2)	N2—C2	1.380 (4)
Gd1—O2^i^	2.375 (2)	N2—H3	0.8600
Gd1—O4^i^	2.407 (3)	N3—C2	1.330 (4)
Gd1—O4	2.407 (3)	N3—H4	0.8600
Gd1—O3^i^	2.414 (2)	N3—H5	0.8600
Gd1—O3	2.414 (2)	O3—H6	0.8067
O1—C1	1.253 (4)	O3—H7	0.7949
O2—C2	1.242 (3)	O4—H8	0.7597
N1—C1	1.314 (4)	O4—H9	0.8018
N1—H1	0.8600		
			
O1^i^—Gd1—O1	126.90 (12)	O4—Gd1—O3	71.88 (8)
O1^i^—Gd1—O2	82.06 (8)	O3^i^—Gd1—O3	127.08 (12)
O1—Gd1—O2	70.41 (7)	C1—O1—Gd1	136.27 (18)
O1^i^—Gd1—O2^i^	70.41 (7)	C2—O2—Gd1	136.83 (18)
O1—Gd1—O2^i^	82.06 (8)	C1—N1—H1	120.0
O2—Gd1—O2^i^	116.04 (11)	C1—N1—H2	120.0
O1^i^—Gd1—O4^i^	75.96 (10)	H1—N1—H2	120.0
O1—Gd1—O4^i^	147.78 (8)	C1—N2—C2	125.1 (3)
O2—Gd1—O4^i^	140.85 (7)	C1—N2—H3	117.4
O2^i^—Gd1—O4^i^	86.53 (9)	C2—N2—H3	117.4
O1^i^—Gd1—O4	147.78 (8)	C2—N3—H4	120.0
O1—Gd1—O4	75.96 (10)	C2—N3—H5	120.0
O2—Gd1—O4	86.53 (9)	H4—N3—H5	120.0
O2^i^—Gd1—O4	140.85 (8)	O1—C1—N1	122.0 (3)
O4^i^—Gd1—O4	95.10 (17)	O1—C1—N2	122.1 (3)
O1^i^—Gd1—O3^i^	129.93 (8)	N1—C1—N2	115.9 (3)
O1—Gd1—O3^i^	75.91 (8)	O2—C2—N3	122.4 (3)
O2—Gd1—O3^i^	144.00 (8)	O2—C2—N2	122.7 (2)
O2^i^—Gd1—O3^i^	70.30 (7)	N3—C2—N2	114.8 (3)
O4^i^—Gd1—O3^i^	71.88 (9)	Gd1—O3—H6	125.2
O4—Gd1—O3^i^	73.11 (8)	Gd1—O3—H7	123.3
O1^i^—Gd1—O3	75.91 (8)	H6—O3—H7	108.2
O1—Gd1—O3	129.93 (8)	Gd1—O4—H8	133.1
O2—Gd1—O3	70.30 (7)	Gd1—O4—H9	131.7
O2^i^—Gd1—O3	144.00 (8)	H8—O4—H9	94.1
O4^i^—Gd1—O3	73.11 (8)		
			
O1^i^—Gd1—O1—C1	81.6 (3)	O4—Gd1—O2—C2	85.5 (3)
O2—Gd1—O1—C1	18.4 (3)	O3^i^—Gd1—O2—C2	30.9 (4)
O2^i^—Gd1—O1—C1	139.7 (3)	O3—Gd1—O2—C2	157.5 (3)
O4^i^—Gd1—O1—C1	−149.9 (3)	Gd1—O1—C1—N1	149.2 (3)
O4—Gd1—O1—C1	−72.9 (3)	Gd1—O1—C1—N2	−31.6 (5)
O3^i^—Gd1—O1—C1	−148.7 (3)	C2—N2—C1—O1	15.1 (5)
O3—Gd1—O1—C1	−21.8 (3)	C2—N2—C1—N1	−165.6 (3)
O1^i^—Gd1—O2—C2	−124.7 (3)	Gd1—O2—C2—N3	161.3 (2)
O1—Gd1—O2—C2	9.2 (3)	Gd1—O2—C2—N2	−21.7 (5)
O2^i^—Gd1—O2—C2	−61.1 (3)	C1—N2—C2—O2	9.9 (5)
O4^i^—Gd1—O2—C2	179.3 (3)	C1—N2—C2—N3	−172.8 (3)

Symmetry codes: (i) −*x*, *y*, −*z*+3/2.

### Hydrogen-bond geometry (Å, °)

**Table:**

*D*—H···*A*	*D*—H	H···*A*	*D*···*A*	*D*—H···*A*
N1—H1···O1^ii^	0.86	2.10	2.910 (4)	157
N1—H2···Cl1^iii^	0.86	2.40	3.194 (3)	154
N2—H3···Cl1^iii^	0.86	2.53	3.315 (3)	153
N3—H4···Cl1^iv^	0.86	2.54	3.363 (3)	161
N3—H5···Cl1^v^	0.86	2.53	3.311 (3)	151
O3—H6···Cl1^vi^	0.81	2.39	3.163 (2)	162
O3—H7···Cl2^vii^	0.79	2.28	3.059 (2)	167
O4—H8···Cl1	0.76	2.45	3.208 (2)	178
O4—H9···Cl2	0.80	2.41	3.127 (2)	150

Symmetry codes: (ii) −*x*+1, *y*, −*z*+3/2; (iii) *x*+1/2, *y*−1/2, *z*; (iv) *x*−1/2, *y*−1/2, *z*; (v) −*x*, −*y*, −*z*+1; (vi) −*x*−1/2, −*y*+1/2, −*z*+1; (vii) *x*−1, *y*, *z*.
